# In Vitro and Ex Vivo Evaluation of Novel Methacrylated Chitosan-PNIPAAm-Hyaluronic Acid Hydrogels Loaded with Progesterone for Applications in Vaginal Delivery

**DOI:** 10.3390/polym16152160

**Published:** 2024-07-30

**Authors:** Oana-Teodora Afloarea, Isabella Nacu, Liliana Vereștiuc, Cătălina Natalia Yilmaz, Alina Diana Panainte, Cătălina Anișoara Peptu, Iulia-Giorgiana Ostafe, Nela Bibire

**Affiliations:** 1 Doctoral School, “Grigore T. Popa” University of Medicine and Pharmacy, 16 Universitatii Street, 700116 Iasi, Romania; oana-teodora_i_lupei@d.umfiasi.ro; 2 Faculty of Medical Bioengineering, Department of Biomedical Sciences, “Grigore T. Popa” University of Medicine and Pharmacy, 16 Universitatii Street, 700116 Iasi, Romania; cobzariu.isabella@icmpp.ro; 3 “Petru Poni” Institute of Macromolecular Chemistry, 41-A Grigore Ghica Voda Alley, 700487 Iasi, Romania; 4 Faculty of Science, Department of Chemistry, Biochemistry Division, Dokuz Eylül University, Kültür Mah. Cumhuriyet Bulv. No:144 Alsancak, 35210 Izmir, Turkey; 5 Faculty of Pharmacy, Department of Analytical Chemistry, “Grigore T. Popa” University of Medicine and Pharmacy, 16 Universitatii Street, 700116 Iasi, Romania; alina-diana.panainte@umfiasi.ro (A.D.P.); nela.bibire@umfiasi.ro (N.B.); 6 Faculty of Chemical Engineering and Environmental Protection “Cristofor Simionescu”, Department of Natural and Synthetic Polymers, “Gheorghe Asachi” Technical University of Iasi, 700050 Iasi, Romania; catipeptu@tuiasi.ro; 7 “Cuza Voda” Obstetrics and Gynecology Clinical Hospital, 34 Cuza Voda Street, 700038 Iasi, Romania; ostafe.iulia-giorgiana@email.umfiasi.ro

**Keywords:** progesterone, mucoadhesion, chitosan methacrylate, hyaluronic acid, NIPAAm, vaginal tissue

## Abstract

Miscarriage is defined as the loss of a pregnancy before 24 weeks and administration of progesterone in pregnancy has considerably decreased the risk of premature birth. Progesterone (PGT) starting from the luteal phase stabilizes pregnancy, promotes differentiation of the endometrium, and facilitates the implantation of the embryo. Within the present study, novel hybrid hydrogels based on chitosan methacrylate (CHT), hyaluronic acid (HA), and poly(*N*-isopropylacrylamide) (PNIPAAm) for vaginal delivery of progesterone were evaluated. The hydrogels were characterized by Fourier transform infrared spectroscopy (FTIR) and scanning electron microscopy (SEM) for structural identity assessment and evaluation of their morphological aspects. The ability to swell, the release capacity, enzymatic degradation, cytotoxicity, and mucoadhesion were also reported. The characterized hydrogels demonstrated mucoadhesive properties in contact with the vaginal tissue of swine and bovine origin as substrates, and biodegradability and controlled release in a simulated vaginal environment. Cytocompatibility tests confirmed the ability of the hydrogels and progesterone to support cell viability and growth. The results showed pH-dependent behavior, controlled drug release, good cytocompatibility, and mucoadhesive properties. The hydrogels with higher chitosan amounts demonstrated better bioadhesive properties. This study provides insights into the potential of these hydrogels for the controlled vaginal delivery of progesterone, with promising therapeutic effects and no cytotoxicity observed. The experimental results indicated that a composition with a moderate content of PNIPAAm was suitable for the controlled delivery of progesterone.

## 1. Introduction

Miscarriage is defined as the loss of a pregnancy before 24 weeks of evolution and upwards of 23 million miscarriages occur each year worldwide, representing 44 miscarriages per minute. Spontaneous abortion is the most common complication of early pregnancy affecting about 15% of clinically recognized pregnancies. This has a substantial impact on the physical and psychological well-being of couples, and the experience of post-traumatic stress disorder [1]. Progesterone (PGT), since the luteal phase, stabilizes pregnancy, promotes differentiation of the endometrium, and facilitates the implantation of the embryo [2]. Further administration of progesterone in pregnancy is necessary to prevent the uterus from expelling the embryo due to its tocolytic effect. According to case studies, taking PGT in pregnancy has considerably decreased the risk of premature birth. Experimental in vitro and ex vivo uterine welds demonstrated the role of PGT in maintaining the relaxation of the uterus and inhibiting inflammation throughout pregnancy [3,4]. This is mainly due to the isomorph PGT-B progesterone. Transcriptional control mediated by the nuclear receiver (progesterone receptor) encodes two isomorphs of PGT, namely, PGT-A and PGT-B. Increased transitions of the level of progesterone at the end of pregnancy are activated by PGT-A, and this promotes the activation of contractility and inflammatory processes; this indicates the maturation and cervical dilation of the uterus, characteristic processes for the pregnancy end, and, implicitly, birth [5,6]. Therefore, treatment with progesterone contributes to maintaining the pregnancy in good conditions, and different drug delivery systems and routes of administration have been tested [7].

Hyaluronic acid (HA) has a complex role in the human body, being involved in hydration, migration, cell proliferation and adhesion, angiogenesis, the formation of granulation tissue and the modulation of osmotic balance, fetal development, regulation of inflammatory processes by interacting with toll-like receptors (TLRs), e.g., TLR4 and TLR2, homeostasis of the environment, and eliminating toxic free radicals from the extracellular environment [8]. HA stimulates the development of macrophages, eosinophils, and epithelial cells. Its anionic character, hydrophilicity, and long chain length allow the absorption of large amounts of water [9,10,11]. HA is a glycosaminoglycan constituted of repeating units of D-glucuronic acid and D-*N*-acetylglucosamine. Hyaluronic acid formulation as a drug delivery system is an attractive option, as hydrogen bonding allows for a large capacity to embed water or biological fluids [12]. The therapeutic effect is increased and the potential side effects are often decreased by its presence. Vaginal administration of hyaluronic acid favors hydration, reduces vaginal dryness, is well tolerated, non-toxic, keeps the vaginal pH normal, and can relieve urogenital symptoms [13].

In recent years, various strategies for developing a hydrogel for vaginal localized therapy have been tested, including chitosan, due to its mimetic features with extracellular biological matrix that provide better tolerability and safety of the biological product used [14]. Chitosan is known as a natural polymer with wound healing, analgesic, fungistatic, antimicrobial, and antioxidant properties, attractive for biomedical, cosmetic, food, and agricultural applications [15]. Chitosan is a polycationic polysaccharide that can be affected by both temperature and pH, swellable due to its hydrophilic groups, enabling the controlled release of the drug [16]. The presence of lysozyme causes the slight degradation of chitosan [17], an important feature when developing a gel for vaginal application.

Among the drawbacks of natural polymers is that they possess a low potential for immunological reactions, inherent biocompatibility, bioactivity, and biodegradability. Additionally, their poor mechanical strength led to their need to be strengthened by chemical modification. Biopolymers can be modified by attaching moieties of semi-synthetic polymers to improve their performance with respect to reactivity, solubility, special self-assembly behavior, responsivity to external stimuli, etc. The chemical functionalization of chitosan with methyl methacrylate has been shown to improve the polymer’s intrinsic mucoadhesive properties, which is an important characteristic for mucosal or topical formulations. This process involves bonding disulfides between acrylate or maleimide groups of polymer and cysteine-rich domains on mucosal surfaces [18,19,20]. Poly(*N*-isopropylacrylamide) (PNIPAAm) is an effective polymer for the preparation of temperature-sensitive hydrogels as it has a lower critical solution temperature (LCST) of 32 °C. PNIPAAm-based synthesized hydrogels undergo a reversible hydrophilic–hydrophobic phase transition around LCST, the hydrogel is in the water absorption state. When the temperature value exceeds the LCST value, the hydrogel depresses and releases most of the water molecules, which is essential in designing an efficient system for releasing active substances at the target site. The vaginal administration of progesterone causes a direct local effect on the vaginal–uterine region by promoting anti-inflammatory effects at the maternal–fetal interface [21,22,23]. 

The aim and motivation of this study was to continue the development of thermosensitive and mucoadhesive vaginal hydrogels based on modified chitosan. Chitosan has been chemically modified using the “grafting” technique to attach PNIPAAm moieties to its side chains. The resulting hydrogels exhibited thermoresponsive behavior and demonstrated potential for the sustained release of progesterone. The present paper proposed additional functionalization of chitosan with hyaluronic acid and PNIPAAm on its side chains to establish an optimal formulation capable of maintaining mucoadhesive properties even after exposure to vaginal fluids. For this purpose, hydrogel formulations with and without progesterone (PGT) were evaluated by the degree of swelling, drug release, enzymatic degradation, in vitro cytocompatibility (human dermal fibroblasts—HDFa), and ex vivo bioadhesion tests on animal vaginal tissue models (swine and bovine originated). Hydrogels based on chitosan methacrylate, hyaluronic acid, and *N*-isopropylacrylamide were specifically designed for the topical delivery of progesterone. The hydrophilic groups of the biopolymers will contribute to the permeation and mucoadhesion of the drug in the vaginal mucosa, while the PNIPAAm polymer facilitates the sol-gel transition of the material and controls the release of the drug. Fang et al. [24] supported the biocompatibility of these polymers with in vivo experiments. Hyaluronic acid contributes to hydration, migration, and cell proliferation; this idea is also highlighted by Qiu et al. [25] and Zhao et al. [26]. The literature discusses various progesterone-loaded formulations that are administered vaginally [27,28,29], but the hydrogels designed have distinctive characteristics thanks to their different processing methods.

Compared to the hydrogels prepared by Fang et al. [24], our hydrogels were synthesized through covalent cross-linking, to ensure the polymer mixture of natural and synthetic components was homogeneous, but also the physical reticulation of hydrogels by the freeze–thaw method. The freeze–thaw method is responsible for the gel’s three-dimensional architecture, and the porous network that develops permits targeted, prolonged, and controlled loading and release of the therapeutic.

## 2. Materials and Methods

### 2.1. Materials

Chitosan (high-molecular-weight chitosan, CsMa, Mw = 310,000–375,000 Da), hyaluronic acid sodium salt (HA) from *Streptococcus equi*, and *N*-isopropylacrylamide were purchased from Sigma-Aldrich (Darmstadt, Germany); ammonium persulfate (APS, purified by recrystallization from methanol), N,N,N′,N′-tetramethylethylenediamine (TEMED), ethanol, and NaOH were purchased from Sigma-Aldrich (Darmstadt, Germany). Phosphate buffer saline (pH = 7.4) was prepared with monosodium phosphate (NaH_2_PO_4_·2H_2_O) and disodium phosphate (Na_2_HPO_4_) from Sigma-Aldrich, Darmstadt, Germany. Chitosan with a 21.2% degree of methacrylation (determined by ^1^H NMR) was prepared using a modified protocol of Camci-Unal et al. [30]. Phosphate buffer saline (pH = 4.5) was prepared with citric acid and HCl from Sigma-Aldrich, Darmstadt, Germany. Thiazolyl blue tetrazolium bromide (MTT), Dulbecco’s Modified Eagle’s Medium–high glucose (DMEM), and Fetal Bovine Serum, were acquired from Sigma-Aldrich, Darmstadt, Germany. Progesterone was gifted by Antibiotice S.A., Iasi, Romania.

The vaginal tissue for the ex vivo experiments was obtained from the TCE 3BRAZI animal farm Zanesti, Neamt, Romania. The vaginal mucosa was carefully cleaned with NaCl and frozen at −20 °C.

The cytocompatibility tests employed specific materials suitable for biological investigations, including Hank’s Balanced Salt Solution (HBSS), Dulbecco’s Modified Eagle Medium (DMEM) HAM F12, Penicillin-Streptomycin-Neomycin (PSN) solution, sterile-filtered fetal bovine serum (FBS) for cell culture, Ethylenediaminetetraacetic acid (EDTA), trypsin, and Thiazolyl Blue Tetrazolium Bromide (MTT), acquired from Sigma Aldrich (Darmstadt, Germany). The chemicals utilized for cell morphology assays included Calcein-acetoxymethyl Ester (Calcein AM), Tetramethylrhodamine Isothiocyanate (TRITC, Rhodamine Phalloidin), and 4′,6-Diamidino-2-Phenylindole, dihydrochloride (DAPI). These reagents were acquired from Sigma Aldrich, Darmstadt, Germany, and Biotium, San Francisco, California, respectively.

### 2.2. Methods

#### Preparation of CHT-HA/PNIPAAm Hydrogels

Chitosan methacrylate solution 1.5 wt.% (pH = 3.5) was obtained by dissolution in acetic acid 1 wt.%. The chitosan solution was adjusted to pH = 7.0 using NaOH and mixed with hyaluronic acid solution 2 wt.% (10% wt:wt). The hydrogels were prepared by mixing the polymer solutions with NIPAAm, at different ratios (Table 1), under continuous stirring at 300 rpm for 2 h. Further, a UV/thermo-labile initiating system constituted from APS (2% mol:mol NIPAAm, as 15 wt.% solution in double-distilled water) and TEMED 15 wt.% solution in double-distilled water was added and homogenized. The APS:TEMED ratio was always constant (mol:mol). The mixtures were transferred into Petri dishes and the reaction was carried out for 4 h at 60 °C. The resulting hydrogels have been purified in double-distilled water with a constant pH (pH ≈ 6.3) to remove the unreacted monomer, initiator, and other residues. Finally, the gels underwent freeze–thaw cycles. The process involved three freeze–thaw cycles at −20 °C for 20 h and then thawing at 25 °C for 4 h.

Progesterone (10 wt.%, against polymeric matrix) was loaded by immersing the lyophilized hydrogels in the drug solution (1 mg/mL in ethanol). The solvent was removed by evaporation at room temperature and the obtained materials were maintained in a lyophilized state in a desiccator until characterization. In Table 1 the obtained formulations are listed.

### 2.3. Characterization Methods

CHT-HA/PNIPAAm hydrogels were evaluated for structural identity and morphology using Fourier-transform infrared spectroscopy (FT-IR) and scanning electron microscopy (SEM). The effectiveness of the prepared formulations was determined after a thorough analysis of the swelling ability, drug delivery capability, enzymatic degradation, cytotoxicity study, and bio-adhesive properties of the vaginal mucosa. 

#### 2.3.1. FTIR Spectroscopy

The structures of CHT-HA/PNIPAAm have been studied and confirmed using Fourier-transform infrared spectroscopy. Using an FTIR spectrometer Platinum—ATR, Berlin, Germany, infrared spectroscopy measurements were carried out by scanning from 4000 to 500 cm^−1^ at a resolution of 2 cm^−1^.

#### 2.3.2. SEM Microscopy

With the use of scanning electron microscopy (SEM) and a Hitachi SU 1510 scanning electron microscope (Tokyo, Japan) the hydrogel’s surface and cross-section morphology were examined. Progesterone-loaded and unloaded CHT-HA/PNIPAAm hydrogels were placed on an aluminum stub and coated with a 7 nm thick coating of gold using Cressington 108 (Cranbrook, BC, Canada) equipment. Ultimately, the SEM images were acquired, and a 25 kV accelerating voltage was implemented for registering the data.

#### 2.3.3. Swelling Properties

In order to examine the pH sensitivity and the interaction between hydrogels and fluids that mimicked the physiological vaginal environment, swelling studies were conducted using the volumetric method on a QIAquickRSpin Column 50 (Thermo Fisher Scientific, New York, NY, USA) device that was fitted with a cellulose-based membrane that limits the passage of the hydrogels. The column was connected to a microsyringe. To attain swelling equilibrium, the materials were submerged in PBS solution with pH values of 4.5 and 7.4 for 90 min at 37 °C in the QIAquickRSpin Column 50 apparatus. For every sample, the buffer solution retention ratio was determined using the following formula at regular intervals:

Q (%) = (W_t_ − W_d_)/W_d_ × 100 (1)

where: W_t_ = W_d_ + V_abs_ (t); W_t_ is the mass of solution absorbed at time t; W_d_ is the mass of the sample; V_ab_s (t) is the volume absorbed at time t. The tests were performed using PBS (T = 37 °C) at pH = 7.4 and pH = 4.5, in triplicate.

#### 2.3.4. Enzymatic Degradation

The in vitro enzymatic degradation behavior of chitosan gels was investigated by using lysozyme (2 × 10^6^ u.i.). Basically, the hydrogels were measured (m = 3 mg) and inserted into the dialysis membrane with a volume of 2 mL of enzyme (concentration 2.4 mg/mL). Subsequently, the samples were submerged in 5 mL of simulated vaginal fluid at pH = 4.5 and were kept in an incubator at 37 °C. Samples were collected at established times for 14 days. The degradation behavior of chitosan was measured spectrophotometrically. The collected solution (0.2 mL) from the analyzed samples was mixed with potassium ferrocyanide (K_3_Fe(CN)_6_) (0.8 mL) in the test tube. Potassium ferrocyanide solution was prepared by dissolving 0.5 g K_3_Fe(CN)_6_ in 0.5 M sodium carbonate (Na_2_CO_3_) to 1 L. The test tube was inserted into the water bath for 15 min at 100 °C. After cooling to room temperature, the solution was diluted to 5 mL with 0.5 M Na_2_CO_3_. The optical absorbance of the solution was measured at 420 nm and a calibration curve from N-acetyl-d-glucosamine was used to calculate the concentration of reduced saccharide.

#### 2.3.5. In Vitro Release Profiles

The release of the drug (progesterone, PGT) from the CHT-HA/PNIPAAm hydrogels was investigated through diffusion experiments by using a dialysis membrane, previously boiled at 100 °C for 1 h in distilled water. The weighed sample and 5 mL of buffer solution were immersed into each dialysis membrane after cooling down.

The dialysis membrane together with the working sample was placed in the centrifuge tube with a volume of 20 mL of buffer solution. The PBS (pH = 7.4) temperature remained constant at 37 °C. A volume of 500 μL of solution was collected at 1 h, 2 h, 4 h, 6 h, 8 h, 24 h, 48 h, and 96 h, and the testing environment was completed with 500 μL of buffer solution. This test was repeated at pH = 4.5, in simulated vaginal fluid. The absorbance of the collected sample was measured at 270 nm using a spectrophotometer. The concentrations were calculated based on the calibration curves measured at the same wavelength. The release pharmacokinetic parameters (k—release rate and *n*—release coefficient) were developed using the Korshmayer–Peppas exponential [31].

#### 2.3.6. Cytocompatibility Tests

Human dermal fibroblast cells (HDFa cell line, primary adult cells, Catalog Number C0135C GIBCO™, Thermo Fisher Scientific, Waltham, MA, USA) were cultured in Dulbecco’s Modified Eagles Medium (DMEM-F12 HAM). The medium was enriched with 10% fetal bovine serum (FBS) and a mixture of 1% penicillin, streptomycin, and neomycin (P/S/N). The cells were subsequently cultured for 24 h at T = 37 °C in the presence of a 5% concentration of carbon dioxide (CO_2_) and a relative humidity of 95%. In order to carry out the cytocompatibility study, 400 µL of full DMEM-F12 HAM was added to each well. Subsequently, a 100 µL cell suspension containing 14 × 10^3^ cells/100 µL was placed in each well (48-well plate). Afterwards, the cells were incubated for 24 h at T = 37 °C in an environment containing 5% CO_2_ in order to promote their adherence to substrate. 

Each hydrogel was sterilized through a 30 min exposure to UV radiation on both sides. The materials were brought into contact with cells and then placed in a 5% CO_2_ environment at T = 37 °C for various durations (24 h, 48 h, 72 h). Furthermore, the wells that did not contain samples, chosen as controls, were refilled with fresh medium to supplement the DMEM-F12 HAM.

In order to perform the MTT test, the samples were taken out from the wells, and the culture media was substituted with a working solution containing MTT (5% MTT in unsupplemented DMEM-F12 HAM culture medium). The cells were exposed to MTT solution for 3 h at T = 37 °C. The released formazan was dissolved in dimethyl sulfoxide (DMSO) at a volume of 500 μL per well and absorbance was quantified at a wavelength of 570 nm using a Tecan Sunrise Plate Reader, Männedorf, Switzerland.

Cell viability was calculated according to Equation (2):

Cell viability (%) = (A_sample_)/(A_control_) × 100 (2)

where A_sample_ is the absorbance of the sample and A_control_ is the absorbance of the negative control. Each result represents the mean viability ± standard deviation of three parallel tests.

Live/dead staining assays were performed using Calcein AM solution, May–Grünwald Giemsa, and DAPI solutions. 

For the calcein test, a 2 µL/mL concentration of Calcein AM solution (Sigma Aldrich, Darmstadt, Germany) in HBBS (with Ca^+2^, Mg^+2^, and without phenol red) was used. The staining process lasted 30 min and, thus, the well was kept in the dark. The images for both methods were taken in phase contrast using a Leica DM IL LED Inverted Microscope with a Phase Contrast System (Leica Microsystems GmbH, Wetzlar, Germany). 

#### 2.3.7. Bioadhesion Tests

The hydrogels were subjected to mucoadhesion testing utilizing a TA.XTplus texture analyzer from StableMicro Systems, Godalming, UK.

Vaginal tissue from pigs and cows was first cleansed, then rinsed with a NaCl solution, and stored at −20 °C in a freezer. The vaginal tissue was set up in simulated vaginal fluid (pH = 4.5) for 15 min at 37 °C after being thawed at room temperature and submerged in SVF. Using double-sided adhesive tape, probes from each matrix were fastened to a cylinder probe with a diameter of 10 mm. The tissue was then in contact for 60 s with the cylinder probe at a force of 0.5 g. Three tests were conducted for each composition, using the pre-boiled dialysis membrane and vaginal tissues that were spun at 500 rpm, T = 37 °C, and V = 200 μL SVF pH = 4.5. 

The generated simulated vaginal fluid has the ability to mimic the physiological environment and facilitate the mucoadhesion process between the hydrogel and the tissue mucosa. At the hydrogel–mucous membrane contact, hydrogen and van der Waals bonds are created, which aid in the ex vivo bioadhesion process. In accordance with the Ethical Committee approval No. 120/31.10.2021, ex vivo bioadhesion studies using swine and bovine tissue were carried out. Using the Exponent program, the hydrogels’ detachment force from tissue and dialysis membrane surfaces, as well as the mechanical work of adhesion (a product of detachment force and distance), were computed. 

##### Preparation of Simulated Vaginal Fluid (SVF)

The primary point of interaction between pathogens and vaginal fluid is the superficial layer of the vaginal epithelium. It has a high glycogen content, and water can be transported into the lowest layers thanks to the thin lipid layer. *Lactobacillus* sp. is present in the vagina and plays the task of metabolizing glycogen in lactic acid to preserve the vaginal environment’s pH acidic. Water (90–95%), mucins, carbohydrates, urea, salts, immunoglobulins, fatty acids, albumin, and other trace elements make up vaginal fluid. The contents of the vaginal simulated fluid include glucose (5 g), glycerol (0.16 g), lactic acid (2 g), acetic acid (1 g), bovine serum albumin (0.018 g), urea (0.4 g), NaCl (3.51 g), KOH (1.4 g), and Ca(OH)_2_ (0.222 g) in 900 mL distilled water [32,33].

## 3. Results and Discussion

**Figure 1:**
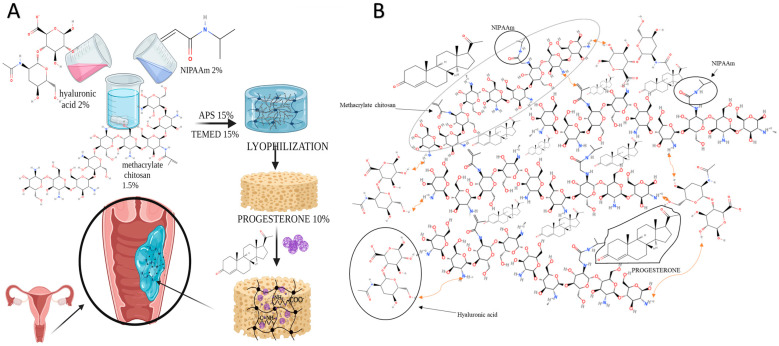
Hybrid hydrogels based on chitosan methacrylate, hyaluronic acid, and PNIPAAm loaded with progesterone: (**A**). Preparation steps. (**B**). The chemical structure of the obtained hydrogel.

Thermally-induced radical polymerization of NIPAAm and crosslinking with chitosan, followed by repeated freeze–thaw cycling (three cycles), were used to generate hybrid hydrogels based on chitosan methacrylate, hyaluronic acid, and poly(*N*-isopropyl acrylamide), either unloaded or loaded with progesterone. The resulting hydrogels were evaluated for their ability to swell, biodegrade, and release drugs under controlled conditions, as well as their cytocompatibility and mucoadhesion, in an ex vivo setting. The progesterone-loaded hydrogel’s production process and molecular structure are depicted in Figure 1A,B.

### 3.1. Hydrogels Structure

**Figure 2:**
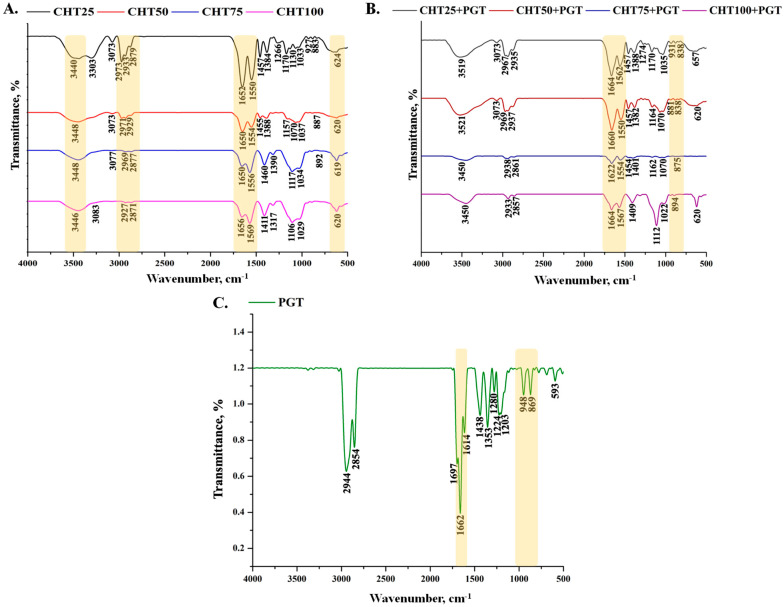
FTIR spectra of hydrogels. (**A**) FTIR spectra of unloaded hydrogels. (**B**) FTIR spectra loaded hydrogels with progesterone. (**C**) FTIR spectra of progesterone.

The FT-IR spectra of CHT loaded/unloaded hydrogels showed peaks at 3440 cm^−1^ and 1652 cm^−1^ which could be assigned to stretching vibration of –NH and –OH, and C=O vibration in the amide group, respectively. The peaks at 2929 cm^−1^ and 2879 cm^−1^ therefore represent the asymmetric and symmetric vibrations of the –CH_2_ groups. Furthermore, the bands at 1388 cm^−1^ and 620 cm^−1^ correspond to the vibration of –CH_2_ bending vibrations. The stretching vibrations of the ester group (C–O–C), characteristic of hyaluronic acid, show stretches between 1022–1074 cm^−1^. The stretching bands at 1068–1020 cm^−1^ are characteristic of a C–O saccharide fragment. In Figure 2A,B, the -NH_2_ stretching peaks of chitosan are shifted from 1550 cm^−1^ to 1569 cm^−1^ due to interactions between –NH_3_^+^ and the -COO^−^ ion groups of hyaluronic acid.

In Figure 2C, the carbonyl (C=O) stretching band at 1662 cm^−1^ served as an indicator for the progesterone’s characteristic absorption bands. Progesterone’s double bond’s distinctive band can be found between 850 cm^−1^ and 900 cm^−1^. The specific band of progesterone for the loaded studied hydrogels was measured at 931 cm^−1^ (CHT25+PGT), 881 cm^−1^ (CHT50+PGT), 875 cm^−1^ (CHT75+PGT), and 894 cm^−1^ (CHT100+PGT) [34,35]. The progesterone was confirmed to be trapped inside the copolymer’s network by the change to lower and/or higher vibration bands, which showed interactions between the polymeric and progesterone’s functional groups.

### 3.2. Hydrogels Morphology

**Figure 3:**
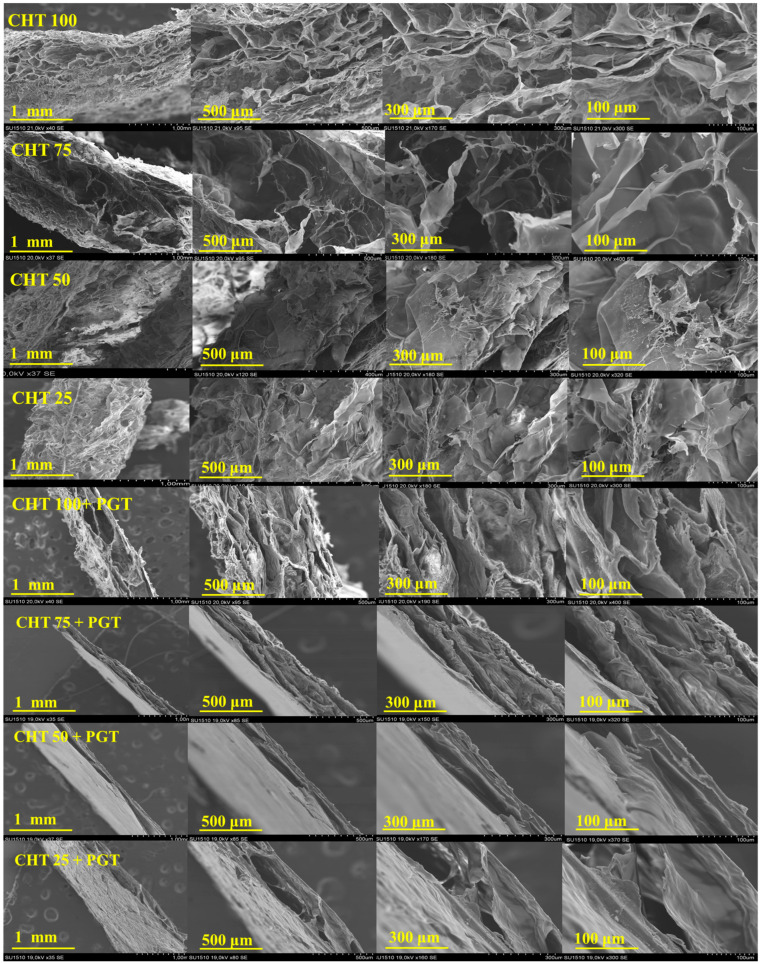
SEM morphology of CHT-HA/PNIPAAm hydrogels unloaded and loaded with progesterone (PGT).

The investigation of the cross-sectional morphology of hydrogels was performed using scanning electron microscopy (SEM). SEM microscopy (Figure 3) confirmed different microstructures depending on the CHT-HA/PNIPAAm ratio. Progesterone-free hydrogels exhibited a homogeneous, sponge-like morphology with interconnected pores. Pore architecture, dimension, and interconnectivity play an important role in hydrogel swelling, drug delivery, controlled degradation kinetics, bio-adhesion, and nutrient diffusion for cell proliferation [36].

The pore size enlarged as the amount of chitosan present in the matrix increased; modified chitosan, with a moderate degree of substitution (21.2% degree of methacrylation, reported to the free amino groups), works as a filler in the 3D network and regulates the interaction with water during the freeze–thawing processes. Porous structure irregularity is attributed to the hyaluronic acid presence, according to Ziminska et al. [37]. Therefore, loading the matrix with progesterone, a layered and more compact structure was observed. The formation of ice crystals and the physical aggregation of polymer chains during the freezing process resulted in crosslinking points. Repeating the freeze–thaw cycles increased the physical crosslink density and formed a three-dimensional matrix, leading to a gel phase. When the ice crystals melted during the thawing phase, a high-porous structure with connections was created [38,39]. Materials loaded with progesterone have layered structures with rough and uneven walls. PGT crystals are uniformly distributed in the polymeric matrix and the SEM images suggest a filling effect of the porous polymeric morphology.

### 3.3. Swelling Properties

The swelling behavior of hydrogels was assessed in PBS solutions with pH values of 7.4 and 4.5. The vaginal environment was reported to have a pH of around 4.0–4.5 [40] and the swelling capacity was expressed as a swelling degree. Both network parameters (interconnectivity, homogeneous distribution of pores, cross-linking density) and the nature of polymers in the composition (hydrophilic polymers) have an impact on the materials’ behavior [41]. Interaction with simulated biological fluids was dependent on the hydrogel composition. As a result, the introduction of hyaluronic acid in hydrogels has improved their water absorption. This difference in the absorption capacity of PBS at the polymer matrices is due to the presence of progesterone within the network. The porous and interconnected structure of the network allows water molecules to be easily infiltrated, in the case of drug-free gels [42]. Chitosan is a polycationic polymer with pKa ≈ 6.2 and the amino groups (NH_3_^+^) on the CHT backbone can be easily protonated in an acidic medium. As such, the hydrogel network is able to absorb more water due to the dissociation of secondary interactions, such as intramolecular hydrogen bonds.

**Figure 4:**
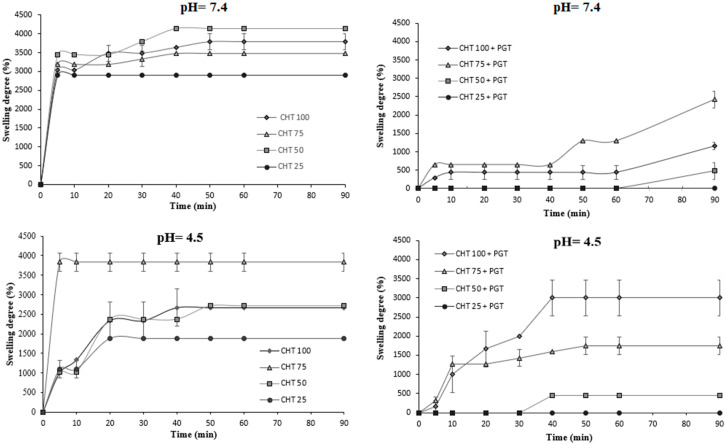
Swelling degree of hydrogels loaded/unloaded progesterone at pH = 4.5 and pH = 7.4.

The presence of progesterone in the matrix influences the swelling behavior of hydrogels, as can be seen in Figure 4.

The swelling of CHT-HA/PNIPAAm hydrogels is caused by the repulsion of charges on the polymer chains and the diffusion of water molecules in the hydrogel pores [23]. Amino groups of chitosan (NH_3_^+^) and carboxyl acid groups (COO^−^) of hyaluronic acid are simultaneously ionized at pH = 4.5 [43]. CHT75 has an abnormal swelling behavior; it may be due to an unhomogeneous network with respect to crosslinking and progesterone distribution in the matrix. When the pH of the solution decreases, electrostatic bonds (NH_3_^+^-COO^−^) between the two polymers (CHT-HA) are formed as both the protonation of the amino groups and the carboxyl groups take place. This effect is not noticeable at higher pH levels due to a reduced amount of deprotonation and repulsion in amino groups of the polymer chains, leading to shrinkage of the complex [44,45]. The enhanced hydrophilicity of ionized groups typically increases water uptake by the polymer matrix, leading to swelling and increased water permeability. Therefore, the amine and carboxyl groups’ ionization regulates the interaction of the matrix with chitosan and vaginal mucosa, especially with the glycoprotein mucin. pH is a key factor for the electrostatic complexation of chitosan and mucin, as the negatively charged mucins interact predominantly electrostatically, resulting in glycoprotein–polysaccharide complexes [46]. The glycoprotein has both hydrophilic and hydrophobic regions with the ability to form electrostatic interactions and H-bonds. The PNIPAM regions in hydrogels will contribute to the H-bonding. The CHT-HA/NIPAAm-based hydrogel shows increased swelling behavior as the pH of this hydrogel equals pKa = 7, promoting electrostatic forces between the mucin and the hydrogel. 

According to the data in Table 2, hydrogels show sensitivity to pH. The drug’s presence in the matrix makes loaded hydrogels more difficult to swell. At pH = 7.4, unloaded materials show higher values than at pH = 4.5, because chitosan has a pKa = 6.2 and tends to swell quickly. Electrostatic interactions at acidic pH allow for the increased absorption of water molecules, which is particularly evident for CHT100+PGT. The increased concentration of NIPAAm prevents the swelling of hydrogels both at pH = 7.4 and at pH = 4.5.

### 3.4. Enzymatic Degradation

Chitosan is enzymatically degradable and can be degraded particularly in the human body by lysozyme. Lysozyme can decompose the β-(1-4)-glycosidic bonds in the methacrylate chitosan backbone. *N*-acetyl-β-D-glucosamine, a degradation product, has been shown to boost fibroblast growth and initiate hyaluronic acid synthesis, thereby aiding in the healing process [47].

**Figure 5:**
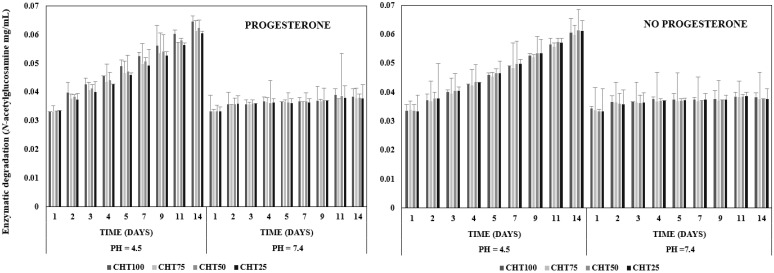
Enzymatic degradation profile for hydrogels loaded/unloaded with progesterone, at pH = 4.5 and pH = 7.4.

Degradation of hydrogels based on chitosan under the action of lysozyme was investigated for 14 days (Figure 5). The results of this study confirmed that all hydrogels loaded/unloaded with progesterone are gradually degraded in the presence of the enzyme, depending on the amount of chitosan they contain. Chitosan degradation by lysozyme tends to be linear at pH = 7.4 and no significant differences in polymer concentrations were observed. The degradation process of chitosan is influenced by the pH of the lysozyme solution. Low pH increases the interaction of amino groups in chitosan with water molecules, as water penetrates the hydrogel matrix causing it to swell. During this hydration process, lysozyme hydrolyzes the specific bond between two *N*-acetylglucosamine units of polysaccharide, causing degradation of chitosan [47,48,49]. 

The degradation rate is more noticeable for hydrogels that contain only chitosan. The degradation behavior of the hydrogels slows down as NIPAAm levels increase. However, at pH = 4.5, a slightly increased degradation for CHT100 can be seen.

The hydrogels maintained their structure even after 14 days of experiment due to the formation of chemical cross-links between the polymers. Agents such as APS and TEMED contributed to the formation of dense, well-cross-linked structures that prevented the degradation of the materials. Thus, the degradation of the covalent bonds formed between the polymer chains requires a much longer time [50].

### 3.5. In Vitro Release Profiles

Progesterone release kinetics confirm the influence of polymer network density on the load/release capacity of the drug product. The experimental results showed a controlled release depending on the composition of the polymer hydrogels and the immersion environment. The initiating system based on APS was used due to low toxicity but also to strengthen the chemical bonds between polymers; the compact structure and porosity of the gel resulted in good control over the release of the drug without it dissolving at pH = 4.5, compared to physically cross-linked hydrogels [41].

**Figure 6:**
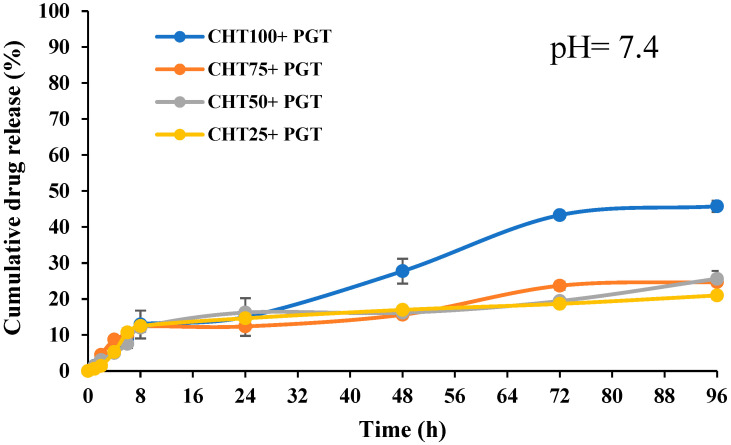
Drug release profiles for CHT100+PGT, CHT75+PGT, CHT50+PGT, and CHT25+PGT at pH = 7.4.

**Figure 7:**
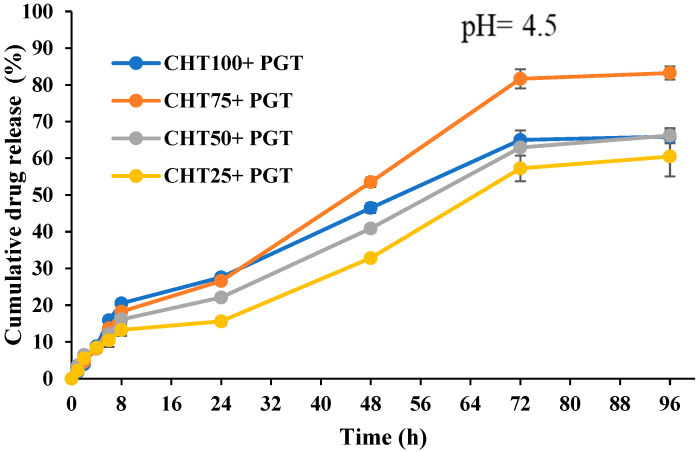
Drug release profiles for CHT100+PGT, CHT75+PGT, CHT50+PGT, and CHT25+PGT at pH = 4.5.

As can be seen in Figure 6 and Figure 7, the release profile of hydrogels at pH = 4.5 showed a better release rate of the drug than at pH = 7.4. As the pH of the solution increases, the amino groups deionize, while the carboxyl groups remain negatively charged. This causes the attraction of Na^+^ ions and allows water diffusion in the complex, but the concentration of Na ions which is too high decreases the difference between the osmotic pressure inside the hydrogel and the ambient solution [51]. Fluctuations in extrinsic parameters, such as ionic strength, pH, and temperature, induce conformation changes. The presence of Na^+^ cations suppresses electrostatic repulsion between carboxylated anions, which together weaken intermolecular hydrogen bonds leading to a decrease in the viscosity of hyaluronic acid and causing hydrophobic behavior of the network.

The polyanion behavior of HA is characterized by the presence of carboxylic acid and primary hydroxyl groups in each disaccharide component; therefore, the polysaccharide is pH-dependent on the reaction medium. The degree of ionization influences the viscoelastic behavior, elasticity, and stability of the hydrogel, as well as the degree of hydration. At physiological pH, HA can be easily complexed with cationic salts due to its negative character being considered a weak polyelectrolyte. The carboxylated anion at pH = 4.0–11.0 is deprotonated (COO^−^), favoring the formation of new intermolecular hydrogen bonds between hydroxyl groups and ether, but also between acetylamine and carboxylate anions [52].

In the first 8 h the phenomenon of “burst effect” is observed, the hormone located on the surface of the polymer matrix is released much faster due to labile interactions with polymer chains. After that, a rapid increase in the release rate of the drug is observed; this is evident at pH = 4.5, where the polymer tends to degrade, the molecular weight decreases, and the matrix becomes more porous due to erosion. Hydration of polymers at pH = 4.5 and electrostatic interactions (NH_3_^+^, COO^−^) favor a porous network in the matrix. However, hydrophobic interactions in the PNIPAAm chains can retain much of the drug, while the diffusion coefficient of the drug indicates a non-Fickian diffusion mechanism according to the equation by Korsmeyer–Peppas (3) [51,53].

(3) $$F=\frac{M_{t}}{M}=k_{m}\;t^{n}$$

where F = fraction of drug release at time “t”; M_t_ = amount of drug released at time “t”; M = total amount of drug in dosage form; k_m_ = kinetic constant; n = diffusion exponent; t = time (h).

Non-Fickian diffusion, also called abnormal diffusion, is a diffusion mechanism that has a time-dependent relationship. This type of mechanism is dependent on the rate of contraction-dependent release of progesterone and the rate of release by progesterone diffusion (Table 3). Progesterone is a small hydrophobic hormone with a mean molecular weight of 314.46 g/mol^−1^, and it is insoluble in water but soluble in ethanol or methanol. Pore size significantly influences the profile and the speed of release of the drug [54]. According to clinical studies, the administration of at least 400 mg/day progesterone is recommended. The administration of Crinone gel shows insufficient serum levels to support the pregnancy (serum absorption level being 9 ng/mL after 24 h). Additionally, Velázquez et al. performed a comparative study and demonstrated that chitosan-based hydrogels had similar progesterone diffusion to that of the commercial Crinone gel formulation. Therefore, prepared materials presented characteristics comparable to those proposed in the literature, while hydrogels can be loaded in order to provide the necessary amount of progesterone to exert its therapeutic effect [35,55].

The Korsmeyer–Peppas model is used to describe the release of progesterone from the polymer matrix system and could involve any or all of the following mechanisms: erosion, diffusion, or swelling. Therefore, the release exponent *n* was used to interpret the diffusion of PGT from the hydrogels [56]. The slope of the graph is in accordance with the ‘*n*’ value. The CHT-HA/PNIPAAm hydrogels had a high correlation coefficient (R_2_ > 0.95). The values of diffusion coefficient *n* at pH = 7.4 correspond to a release mechanism of the drug by diffusion (Fickian diffusion, *n* < 0.45) (Table 3) [57]. At pH = 4.5, the diffusion kinetics of the hydrogels follow almost faithfully the non-Fickian diffusion model (0.5 < *n* < 0.89) and the swelling-controlled mechanism, according to the correlation coefficient (R_2_ > 0.98) [58]. 

In the meanwhile, PNIPAAm chains have an important role in deterring the medication from diffusing too rapidly. The most investigated and utilized smart polymer for drug release management is PNIPAAm. It is well recognized for its vulnerability to variations in temperature in addition to its hydrophobic and hydrophilic characteristics, which facilitate a shift in the glob-to-coil conformation, thereby making it easier to transition from a dehydrated to a hydrated state. PNIPAAm is a neutral amphiphilic molecule that contains hydrophilic alcohol, ether, or amide groups (–CONH–), and hydrophobic hydrocarbon backbone chains (the isopropyl groups –CH(CH_3_)_2_) [59]. As PNIPAAm chains and water undergo hydrogen bond contraction over LCST, PNIPAAm loses its hydrated state and becomes insoluble [57]. The temperature rise induced a hydrophilic/hydrophobic equilibrium, enabling it to provide a steady and regulated release of the hormone. The reduced CHT-HA/PNIPAAm ratio of 75/25 at CHT-HA/PNIPAAm 25/75 reduces the hydrogel’s capacity to absorb water and retain water, which hinders the development and expansion of the pores that are required for drug diffusion [60].

### 3.6. Cytocompatibility Tests

Progesterone is a steroid hormone involved mainly in maintaining pregnancy, due to its anti-inflammatory effect. According to research, the low oral bioavailability of PGT is due to its low solubility in water and in aqueous solution (7 pg/mL at T = 25 °C), but also due to extensive first-pass hepatic metabolism [61]. Researchers have tried to create various formulas that facilitate the solubility of PGT, for example, micronized oil-based progesterone. However, the bioavailability of the drug failed to exceed the 10% threshold, thus requiring higher doses of 200 mg/day and 300 mg/day, in some cases resulting in low patient tolerance [62]. Vaginal administration of progesterone can be a more efficient delivery method than oral administration due to the uterine first-pass effect, a process including the drug transport from the vagina to the uterus through the uterovaginal plexus. In a recent study, Miles et al. found an increased level of progesterone in the vaginal tissue than after intramuscular administration [63]. To enhance understanding of the absorption mechanism of drugs at the vaginal level, Cicinelli et al. used ^99m^Tc-pertechnetat (Na^+99m^TcO_4_^−^) radionuclide as a tracer. Its absorption in vivo demonstrated the occurrence of a countercurrent vascular exchange between the vagina and the uterus due to the uterovaginal venous plexus. This study strongly supports vaginal drug delivery as an effective route for progesterone and other drugs [64].

**Figure 8:**
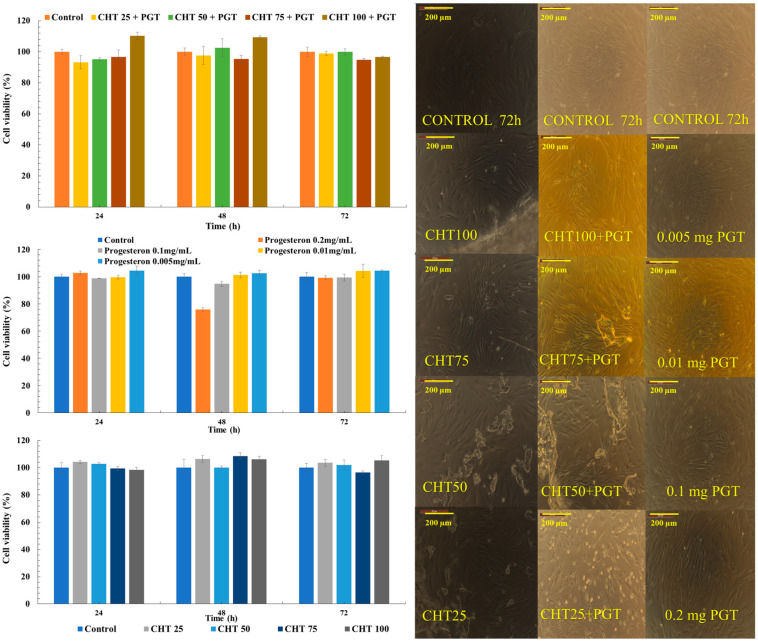
NHDF viability data from the MTT assays.

Therefore, after analyzing the mechanism by which a drug penetrates the systemic pathway, using the MTT method for cell viability, we investigated the effect of progesterone on fibroblasts, but also the biocompatibility of the hydrogels applied to the cells (Figure 8).

**Figure 9:**
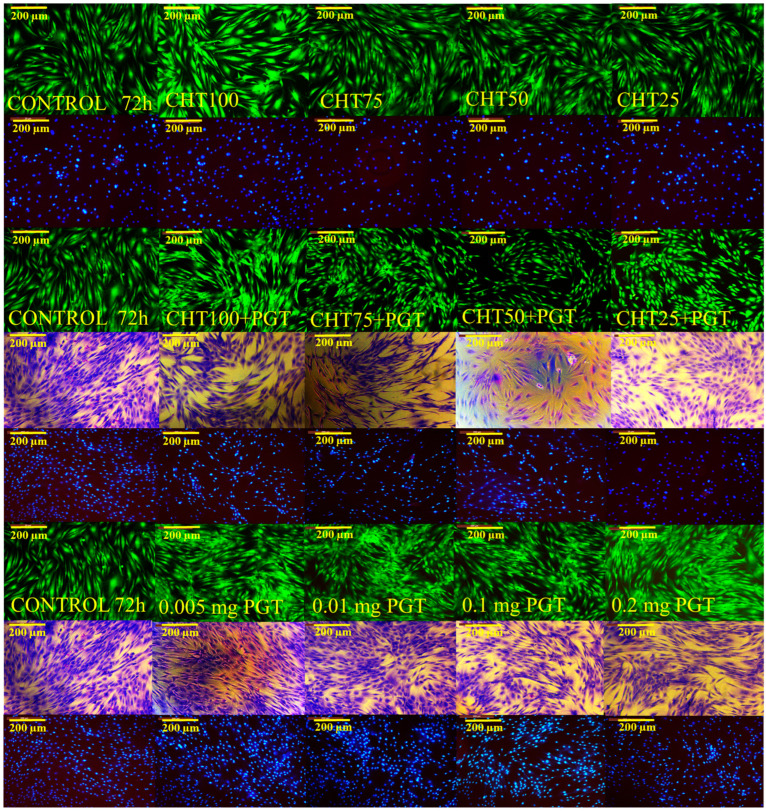
Viable cells and the morphology appearance after the colorings with Calcein AM, May–Grünwald Giemsa, and DAPI, after 72 h of incubation.

Microscopic images describe the morphological appearance of living cells. Progesterone supported cell growth but changed their morphological appearance. Some of the fibroblasts are more elongated, while others appear thickened, and, nevertheless, the cells proliferate and interact with each other. However, fibroblasts are adherent to substrate, contoured, and have a uniform monolayer, but their appearance changes in the presence of progesterone. The plates were stained with May–Grünwald Giemsa, Calcein AM, and DAPI to study cell morphology in detail, with regard to cytoplasmic details, cell size, and cell nucleus (Figure 9) [65].

Increasing the concentration of progesterone (0.005 mg, 0.01 mg, 0.1 mg, 0.2 mg) does not induce toxicity, and cell viability is conserved. However, as the concentration of PGT increases, the morphology of the fibroblasts changes to a thickened appearance. The hydrogels do not induce a cytotoxic effect, which proves that the polymers are biocompatible with human fibroblasts. In cervical tissue, the predominant cells are fibroblasts, and, consequently, it is essential that the dosage forms support cell proliferation. In a study on human cervical fibroblasts, researchers reported that progesterone hydrogels significantly promoted increased cell adhesion [66]. Moreover, Cappela et al. demonstrated that NIPAAm-based hydrogels are non-toxic and non-genotoxic. Consistent with previously obtained results, Rivero et al. demonstrated that NIPAAm gels have no negative effect on the mitochondrial activity of bovine fetal fibroblasts [67]. Another important aspect is the intracellular activity of hyaluronic acid, which supports cell migration, differentiation, and proliferation at the cellular level [68]. In the majority of studies reported, chitosan shows minimal toxic effects, thus justifying its selection as a safe biomaterial in drug delivery.

However, it is essential to emphasize safety studies when using chitosan derivatives for pharmaceutical or biomedical use. In conclusion, progesterone hydrogels increase cell viability; therefore, both progesterone and formulations do not exhibit cytotoxic effects.

### 3.7. Bioadhesion Tests

The mechanism of bio-adhesion between the vaginal mucosa and hydrogel is characterized by the interaction between mucin and the ionizable groups of polymers. The use of hybrid hydrogels, based on natural polymers and synthetic ones, is aimed at developing a pharmaceutical form with a long residence time and a sustained drug therapeutic effect. Mucoadhesion ensures drug absorption and release at the mucus epithelium’s surface.

The mechanism of mucoadhesion is closely correlated with the water absorption capacity. The swelling involves the entanglement and subsequent interpenetration of the mucin chains in the inflated polymer matrix, while chemical interactions comprise hydrogen bonds and electrostatic bonds between the positively charged amino groups in CHT and the negatively charged components of the mucus layer [69]. Bioadhesion tests were performed ex vivo on swine and bovine vaginal tissue immersed in SVF to achieve the conditions of a human vaginal environment. Intracellular delivery of the drug requires the penetration of mucus. The residence time in the vaginal environment is limited due to the self-cleaning mechanism, mucus is secreted, eliminated, and continuously renewed. Mucus’s primary component is mucin, which is made up of a polypeptide backbone that has repeating domains of proline (P), threonine (T), and serine (S), which are strongly O-glycosylated by T and the residues of S [46,70,71,72].

Electrostatic interactions between negatively charged mucin and cationic groups of chitosan cause the formation of intimate contact between the hydrogel and the mucosa [73]. Therefore, the interlocking chains of CHT-HA with mucin favor electron transfer, forming an electric double layer at the polymer/mucin interface. This mechanism increases the bioavailability of the drug due to ionizable groups with tight epithelial junctions, which allow the penetration of the drug into the mucosa [71,74]. Additionally, PNIPAAm plays an important role in the gel composition, which allows the regulation of mucoadhesion and improves the resistance time of the dosage form. Over LCST, the hydrophobicity of the polymer increases, and the water molecules separate from the polymer chain and therefore lose their elastic stretch morphology [75]. Peppas and Huang demonstrated that hydrophobicity plays an important role in the mucoadhesion process [76]. By the so-called hydrophobic effect, they theorized that mucins can be absorbed on hydrophobic surfaces, due to the van der Waals forces between water molecules and the hydrophobic macromolecule. This situation is determined by the loss of entropy of water molecules that are gradually associated with hydrophobic groups of polymers [77]. During experiments, they discovered the possibility of hydrophobic interactions between chitosan and mucin [78]. However, the association between the mucosa and hydrophobic groups does not reflect a significant increase in mucoadhesion. 

**Figure 10:**
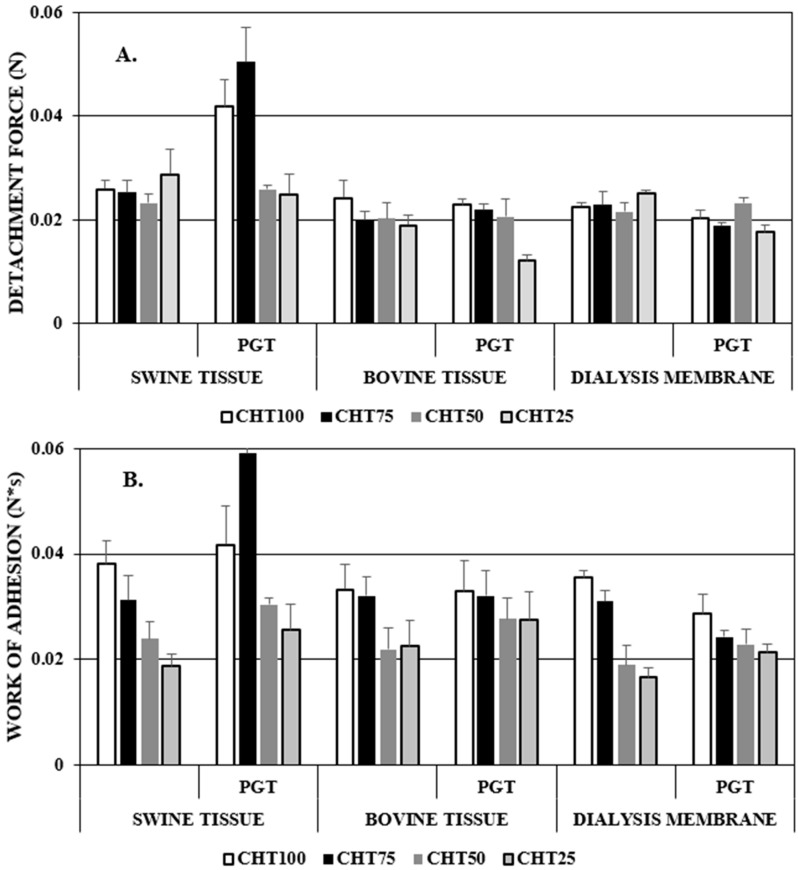
Bioadhesive parameters for free and progesterone-loaded hydrogels: (**A**) detachment force (N) at pH = 4.5; (**B**) work of adhesion (N*s) at pH = 4.5.

The amount of CHT polymer in the hydrogel influenced the mechanical work of adhesion (the product between detachment and distance) and the material detachment force on the contact surface, as can be seen in Figure 10. 

Cross-linked materials with an increased amount of PNIPAAm have lower adhesion values compared to materials containing a higher amount of chitosan. The adhesion force increases with the increase in the adhesive group content in the hydrogel’s composition (carboxyl groups, hydroxyl groups, amino groups) and with the speed of absorption of aqueous solutions. Materials that were more adhesive to swine vaginal tissue showed greater potential for mucoadhesion compared to bovine vaginal tissue and dialysis membrane, and the results are determined by tissue architectures and mucosa characteristics.

The results suggested that the hydrogels CHT100+PGT and CHT75+PGT have much better bioadhesive properties compared to other materials. The combination of thermosensitive polymers with natural bioadhesive polymers permanently favors the vaginal mucosa for a longer period of time for the release of hydrophobic drugs through the mucosa [79]. 

## 4. Conclusions

This paper provides an important insight for understanding the impact of vaginal pH on the permeability and performance of hydrogels based on chitosan methacrylate, hyaluronic acid, and PNIPAAm, fabricated by free-radical polymerization, and loaded with progesterone. It was found that pH transitions from 4.5 to 7.4 substantially affected the degree of swelling, diffusion, and release of the drug, as well as enzymatic degradation of the hybrid hydrogels. The more acidic vaginal environment favored the swelling and adhesion of the hydrogel CHT-HA/NIPAAm 75/25 and CHT-HA to the vaginal tissue. Bioadhesive behavior promotes drug residence time with mucosal tissue, which can lead to the controlled absorption of progesterone. Cytocompatibility studies with MTT have demonstrated that prepared hybrid hydrogels do not induce cytotoxic effects. However, biopolymers help with cell differentiation and migration. The promising therapeutic effect of hydrogels loaded with PGT is supported by progesterone concentrations without causing any adverse effects. Consequently, hydrogels are suitable for supporting cells and delivering drugs to target sites for therapeutic effects.

This study has potential limitations related to the release of progesterone content in the pathological vaginal environment. However, further investigations will be conducted to enhance the concentration of the drug released in the acidic vaginal environment.

## Figures and Tables

**Table 1:** The list of obtained formulations.

COD	CHITOSAN wt/wt %	NIPAAm wt/wt %	Formulation
CHT 100	100	0	The polymer solution (CHT-HA) was mixed with NIPAAm and the initiator solutions, for 4 h at 60 °C, purification and freeze-thaw cycling (3 cycles), hydrogels lyophilization.
CHT 75	75	25	
CHT 50	50	50	
CHT 25	25	75	
CHT 100 PGT	100	0	The polymer solution (CHT-HA) was mixed with NIPAAm and the initiator solutions, 4 h 60 °C, purification and freeze-thaw cycling (3 cycles). Finally, the hydrogels were lyophilized and loaded with 10% progesterone.
CHT 75 PGT	75	25	
CHT 50 PGT	50	50	
CHT 25 PGT	25	75	

**Table 2:** Swelling degree of hydrogels at pH = 7.4 and pH = 4.5.

pH	CHT100	CHT75	CHT50	CHT25	CHT100+PGT	CHT75+PGT	CHT50+PGT	CHT25+PGT
7.4	3788%	3478%	4138%	2899%	1159%	2903%	1270%	615%
4.5	2667%	3833%	2712%	1875%	3000%	1746%	455%	0%

**Table 3:** Model constants and R-squared of progesterone diffusion profile from hydrogels fitted to Korsmeyer–Peppas model.

pH 4.5	CHT25+PGT	CHT50+PGT	CHT75+PGT	CHT100+PGT	pH 7.4	CHT25+PGT	CHT50+PGT	CHT75+PGT	CHT100+PGT
k	0.194	0.351	0.351	0.434	**k**	0.22	0.381	0.32	0.368
*n*	0.744	0.628	0.71	0.589	***n***	0.655	0.386	0.423	0.369
R_2_	0.984	0.988	0.99	0.99	**R_2_**	0.988	0.966	0.97	0.95

## Data Availability

The data presented in this study are available on request from the corresponding authors.

## References

[1] Romero R., Conde-Agudelo A., Da Fonseca E., O’Brien J.M., Cetingoz E., Creasy G.W., Hassan S.S., Nicolaides K.H. (2018). Vaginal Progesterone for Preventing Preterm Birth and Adverse Perinatal Outcomes in Singleton Gestations with a Short Cervix: A Meta-Analysis of Individual Patient Data. Am. J. Obstet. Gynecol..

[2] Vaisbuch E., Leong M., Shoham Z. (2012). Progesterone Support in IVF: Is Evidence-Based Medicine Translated to Clinical Practice? A Worldwide Web-Based Survey. Reprod. Biomed. Online.

[3] Sroussi J., Bourret A., Pourcelot A.-G., Thubert T., Lesavre M., Legendre G., Tuffet S., Rousseau A., Benifla J.-L. (2022). Does Hyaluronic Acid Gel Reduce Intrauterine Adhesions after Dilation and Curettage in Women with Miscarriage? A Multicentric Randomized Controlled Trial (HYFACO Study). Am. J. Obstet. Gynecol..

[4] Tripathy S., Nallasamy S., Mahendroo M. (2022). Progesterone and Its Receptor Signaling in Cervical Remodeling: Mechanisms of Physiological Actions and Therapeutic Implications. J. Steroid Biochem. Mol. Biol..

[5] Sugita Y., Kuwabara Y., Katayama A., Matsuda S., Manabe I., Suzuki S., Oishi Y. (2023). Characteristic Impairment of Progesterone Response in Cultured Cervical Fibroblasts Obtained from Patients with Refractory Cervical Insufficiency. Sci. Rep..

[6] Miyazaki K., Dyson M.T., Coon V.J.S., Furukawa Y., Yilmaz B.D., Maruyama T., Bulun S.E. (2018). Generation of Progesterone-Responsive Endometrial Stromal Fibroblasts from Human Induced Pluripotent Stem Cells: Role of the WNT/CTNNB1 Pathway. Stem Cell Rep..

[7] Sundström-Poromaa I., Comasco E., Sumner R., Luders E. (2020). Progesterone—Friend or Foe?. Front. Neuroendocrinol..

[8] Jiang D., Liang J., Noble P.W. (2011). Hyaluronan as an Immune Regulator in Human Diseases. Physiol. Rev..

[9] Sudha P.N., Rose M.H. (2014). Beneficial Effects of Hyaluronic Acid. Advances in Food and Nutrition Research.

[10] Bai Q., Gao Q., Hu F., Zheng C., Chen W., Sun N., Liu J., Zhang Y., Wu X., Lu T. (2023). Chitosan and Hyaluronic-Based Hydrogels Could Promote the Infected Wound Healing. Int. J. Biol. Macromol..

[11] Buckley C., Murphy E.J., Montgomery T.R., Major I. (2022). Hyaluronic Acid: A Review of the Drug Delivery Capabilities of This Naturally Occurring Polysaccharide. Polymers.

[12] Kikani T., Dave S., Thakore S. (2023). Functionalization of Hyaluronic Acid for Development of Self-Healing Hydrogels for Biomedical Applications: A Review. Int. J. Biol. Macromol..

[13] Caramella C.M., Rossi S., Ferrari F., Bonferoni M.C., Sandri G. (2015). Mucoadhesive and Thermogelling Systems for Vaginal Drug Delivery. Adv. Drug Deliv. Rev..

[14] Prado G.H.C., Prado I.M. (2021). Hydrogels Based on Natural Polysaccharides and Their Applications. Comprehensive Glycoscience.

[15] Li X., Yang Y., Yang F., Wang F., Li H., Tian H., Wang G. (2021). Chitosan Hydrogel Loaded with Recombinant Protein Containing Epitope C from HSP90 of Candida Albicans Induces Protective Immune Responses against Systemic Candidiasis. Int. J. Biol. Macromol..

[16] Luo C., Guo A., Zhao Y., Sun X. (2022). A High Strength, Low Friction, and Biocompatible Hydrogel from PVA, Chitosan and Sodium Alginate for Articular Cartilage. Carbohydr. Polym..

[17] Lončarević A., Ivanković M., Rogina A. (2017). Lysozyme-Induced Degradation of Chitosan: The Characterisation of Degraded Chitosan Scaffolds. J. Tissue Repair Regen..

[18] Talaei F., Azhdarzadeh M., Hashemi Nasel H., Moosavi M., Foroumadi A., Dinarvand R., Atyabi F. (2011). Core Shell Methyl Methacrylate Chitosan Nanoparticles: In Vitro Mucoadhesion and Complement Activation. Daru J. Fac. Pharm. Tehran Univ. Med. Sci..

[19] Kolawole O.M., Lau W.M., Khutoryanskiy V.V. (2018). Methacrylated Chitosan as a Polymer with Enhanced Mucoadhesive Properties for Transmucosal Drug Delivery. Int. J. Pharm..

[20] Jaiswal S., Dutta P.K., Kumar S., Koh J., Pandey S. (2019). Methyl Methacrylate Modified Chitosan: Synthesis, Characterization and Application in Drug and Gene Delivery. Carbohydr. Polym..

[21] Kang S.M., Lim S., Choi J.S. (2022). On the Nature of Wetting Transition on High-Aspect-Ratio pNIPAAm Micropillar Structures. Surf. Interfaces.

[22] Qin Z., Zhang R., Xu Y., Cao Y., Xiao L. (2021). A One-Pot Synthesis of Thermosensitive PNIPAAM Interpenetration Polymer Networks(IPN) Hydrogels. JCIS Open.

[23] Puleo G.L., Zulli F., Piovanelli M., Giordano M., Mazzolai B., Beccai L., Andreozzi L. (2013). Mechanical and Rheological Behavior of pNIPAAM Crosslinked Macrohydrogel. React. Funct. Polym..

[24] Fang J. (2008). Temperature-Sensitive Hydrogels Composed of Chitosan and Hyaluronic Acid as Injectable Carriers for Drug Delivery. Eur. J. Pharm. Biopharm..

[25] Qiu Y., Yang T., Zhang H., Dai H., Gao H., Feng W., Xu D., Duan J. (2024). The Application of pH-Responsive Hyaluronic Acid-Based Essential Oils Hydrogels with Enhanced Anti-Biofilm and Wound Healing. Int. J. Biol. Macromol..

[26] Zhao Y., Liu X., Peng X., Zheng Y., Cheng Z., Sun S., Ding Q., Liu W., Ding C. (2022). A Poloxamer/Hyaluronic Acid/Chitosan-Based Thermosensitive Hydrogel That Releases Dihydromyricetin to Promote Wound Healing. Int. J. Biol. Macromol..

[27] Almomen A., Cho S., Yang C.-H., Li Z., Jarboe E.A., Peterson C.M., Huh K.M., Janát-Amsbury M.M. (2015). Thermosensitive Progesterone Hydrogel: A Safe and Effective New Formulation for Vaginal Application. Pharm. Res..

[28] Shapiro R.L., Bockley K.M., Hsueh H.T., Appell M.B., Carter D.M., Ortiz J., Brayton C., Ensign L.M. (2024). Hypotonic, Gel-Forming Delivery System for Vaginal Drug Administration. J. Control. Release.

[29] Nie L., Zou P., Dong J., Sun M., Ding P., Han Y., Ji C., Zhou Q., Yuan H., Suo J. (2019). Injectable Vaginal Hydrogels as a Multi-Drug Carrier for Contraception. Appl. Sci..

[30] Camci-Unal G., Cuttica D., Annabi N., Demarchi D., Khademhosseini A. (2013). Synthesis and Characterization of Hybrid Hyaluronic Acid-Gelatin Hydrogels. Biomacromolecules.

[31] Wu I.Y., Bala S., Škalko-Basnet N., Di Cagno M.P. (2019). Interpreting Non-Linear Drug Diffusion Data: Utilizing Korsmeyer-Peppas Model to Study Drug Release from Liposomes. Eur. J. Pharm. Sci..

[32] Knuth K., Amiji M., Robinson J.R. (1993). Hydrogel Delivery Systems for Vaginal and Oral Applications. Adv. Drug Deliv. Rev..

[33] Tester R., Al-Ghazzewi F.H. (2018). Intrinsic and Extrinsic Carbohydrates in the Vagina: A Short Review on Vaginal Glycogen. Int. J. Biol. Macromol..

[34] Liu Q., Wang X., Zhang H. (2007). Solvent Effects on Infrared Spectra of Progesterone in CHCl3/Cyclo-C6H12 Binary Solvent Systems. Spectrochim. Acta. A Mol. Biomol. Spectrosc..

[35] Velázquez N.S., Turino L.N., Luna J.A., Mengatto L.N. (2019). Progesterone Loaded Thermosensitive Hydrogel for Vaginal Application: Formulation and in Vitro Comparison with Commercial Product. Saudi Pharm. J..

[36] Koohzad F., Asoodeh A. (2024). Development of a Highly Porous Bioscaffold by the Combination of Bubble Entrapping and Freezing-Thawing Techniques to Fabricate Hyaluronic Acid/Gelatin Tri-Layer Wound Dressing. Int. J. Biol. Macromol..

[37] Ziminska M., Wilson J.J., McErlean E., Dunne N., McCarthy H.O. (2020). Synthesis and Evaluation of a Thermoresponsive Degradable Chitosan-Grafted PNIPAAm Hydrogel as a “Smart” Gene Delivery System. Materials.

[38] Waresindo W.X., Luthfianti H.R., Priyanto A., Hapidin D.A., Edikresnha D., Aimon A.H., Suciati T., Khairurrijal K. (2023). Freeze–Thaw Hydrogel Fabrication Method: Basic Principles, Synthesis Parameters, Properties, and Biomedical Applications. Mater. Res. Express.

[39] Qian L., Zhang H. (2011). Controlled Freezing and Freeze Drying: A Versatile Route for Porous and Micro-/Nano-Structured Materials. J. Chem. Technol. Biotechnol..

[40] Das Purkayastha S., Bhattacharya M.K., Prasad H.K., De Mandal S. (2020). Diversity and the Antimicrobial Activity of Vaginal Lactobacilli: Current Status and Future Prospective. Recent Advancements in Microbial Diversity.

[41] Maiz-Fernández S., Barroso N., Pérez-Álvarez L., Silván U., Vilas-Vilela J.L., Lanceros-Mendez S. (2021). 3D Printable Self-Healing Hyaluronic Acid/Chitosan Polycomplex Hydrogels with Drug Release Capability. Int. J. Biol. Macromol..

[42] Afloarea O.-T., Cheaburu Yilmaz C.N., Verestiuc L., Bibire N. (2022). Development of Vaginal Carriers Based on Chitosan-Grafted-PNIPAAm for Progesterone Administration. Gels.

[43] Maiz-Fernández S., Pérez-Álvarez L., Silván U., Vilas-Vilela J.L., Lanceros-Mendez S. (2022). Photocrosslinkable and Self-Healable Hydrogels of Chitosan and Hyaluronic Acid. Int. J. Biol. Macromol..

[44] Ritt C.L., Werber J.R., Wang M., Yang Z., Zhao Y., Kulik H.J., Elimelech M. (2020). Ionization Behavior of Nanoporous Polyamide Membranes. Proc. Natl. Acad. Sci. USA.

[45] Polexe R., Delair T. (2013). Elaboration of Stable and Antibody Functionalized Positively Charged Colloids by Polyelectrolyte Complexation between Chitosan and Hyaluronic Acid. Molecules.

[46] Collado-González M., González Espinosa Y., Goycoolea F.M. (2019). Interaction Between Chitosan and Mucin: Fundamentals and Applications. Biomimetics.

[47] Ren D., Yi H., Wang W., Ma X. (2005). The Enzymatic Degradation and Swelling Properties of Chitosan Matrices with Different Degrees of N-Acetylation. Carbohydr. Res..

[48] Zhao B., Zhao M., Sun H., Yang Y., Sun S., Yu H., He M., Sun Y., Cheng Y. (2022). Preparation and Characterization of Photo-Oxidative Dual-Crosslinked Chitosan/Hyaluronic Acid Hydrogels. React. Funct. Polym..

[49] Zhu L., Bratlie K.M. (2018). pH Sensitive Methacrylated Chitosan Hydrogels with Tunable Physical and Chemical Properties. Biochem. Eng. J..

[50] El Idrissi A., Channab B., Essamlali Y., Zahouily M. (2024). Superabsorbent Hydrogels Based on Natural Polysaccharides: Classification, Synthesis, Physicochemical Properties, and Agronomic Efficacy under Abiotic Stress Conditions: A Review. Int. J. Biol. Macromol..

[51] Argin S., Kofinas P., Lo Y.M. (2014). The Cell Release Kinetics and the Swelling Behavior of Physically Crosslinked Xanthan–Chitosan Hydrogels in Simulated Gastrointestinal Conditions. Food Hydrocoll..

[52] Casey-Power S., Ryan R., Behl G., McLoughlin P., Byrne M.E., Fitzhenry L. (2022). Hyaluronic Acid: Its Versatile Use in Ocular Drug Delivery with a Specific Focus on Hyaluronic Acid-Based Polyelectrolyte Complexes. Pharmaceutics.

[53] Busatto C., Pesoa J., Helbling I., Luna J., Estenoz D. (2018). Effect of Particle Size, Polydispersity and Polymer Degradation on Progesterone Release from PLGA Microparticles: Experimental and Mathematical Modeling. Int. J. Pharm..

[54] Zhang Y., Shams T., Harker A.H., Parhizkar M., Edirisinghe M. (2018). Effect of Copolymer Composition on Particle Morphology and Release Behavior in Vitro Using Progesterone. Mater. Des..

[55] Chantilis S.J., Zeitoun K.M., Patel S.I., Johns D.A., Madziar V.A., McIntire D.D. (1999). Use of Crinone∗ Vaginal Progesterone Gel for Luteal Support in in Vitro Fertilization Cycles. Fertil. Steril..

[56] Lavanya M., Jeyakumar S., Veerappa V.G., Pushpadhas H.A., Ramesha K.P., Kumaresan A., Manimaran A., Eljeeva Emerald F.M. (2024). Fabrication and Characterization of Progesterone Loaded Pullulan Nanofibers for Controlled Release. J. Drug Deliv. Sci. Technol..

[57] Kim A.R., Lee S.L., Park S.N. (2018). Properties and in Vitro Drug Release of pH- and Temperature-Sensitive Double Cross-Linked Interpenetrating Polymer Network Hydrogels Based on Hyaluronic Acid/Poly (N-Isopropylacrylamide) for Transdermal Delivery of Luteolin. Int. J. Biol. Macromol..

[58] Olejnik A., Kapuscinska A., Schroeder G., Nowak I. (2017). Physico-Chemical Characterization of Formulations Containing Endomorphin-2 Derivatives. Amino Acids.

[59] Shaibie N.A., Ramli N.A., Mohammad Faizal N.D.F., Srichana T., Mohd Amin M.C.I. (2023). Poly( *N* -isopropylacrylamide)-Based Polymers: Recent Overview for the Development of Temperature-Responsive Drug Delivery and Biomedical Applications. Macromol. Chem. Phys..

[60] Li G., Guo L., Chang X., Yang M. (2012). Thermo-Sensitive Chitosan Based Semi-IPN Hydrogels for High Loading and Sustained Release of Anionic Drugs. Int. J. Biol. Macromol..

[61] Kolatorova L., Vitku J., Suchopar J., Hill M., Parizek A. (2022). Progesterone: A Steroid with Wide Range of Effects in Physiology as Well as Human Medicine. Int. J. Mol. Sci..

[62] Chen X., Partheniadis I., Nikolakakis I., Al-Obaidi H. (2020). Solubility Improvement of Progesterone from Solid Dispersions Prepared by Solvent Evaporation and Co-Milling. Polymers.

[63] Yang Z., Wu X., Wang H., Zhou J., Lin X., Yang P. (2024). Vagina, a Promising Route for Drug Delivery. J. Drug Deliv. Sci. Technol..

[64] Cicinelli E., Rubini G., De Ziegler D., Barba B., Pinto V., Di Stefano M.G., Mele M. (2001). Absorption and Preferential Vagina-to-Uterus Distribution after Vaginal Administration of 99mTc-Pertechnetate in Postmenopausal Women. Fertil. Steril..

[65] Nacu I., Bercea M., Niță L.E., Peptu C.A., Butnaru M., Vereștiuc L. (2023). 3D Bioprinted Scaffolds Based on Functionalized Gelatin for Soft Tissue Engineering. React. Funct. Polym..

[66] Shukla V., Barnhouse V., Ackerman W.E., Summerfield T.L., Powell H.M., Leight J.L., Kniss D.A., Ghadiali S.N. (2018). Cellular Mechanics of Primary Human Cervical Fibroblasts: Influence of Progesterone and a Pro-Inflammatory Cytokine. Ann. Biomed. Eng..

[67] Capella V., Rivero R.E., Liaudat A.C., Ibarra L.E., Roma D.A., Alustiza F., Mañas F., Barbero C.A., Bosch P., Rivarola C.R. (2019). Cytotoxicity and Bioadhesive Properties of Poly-N-Isopropylacrylamide Hydrogel. Heliyon.

[68] Hwang H.S., Lee C.-S. (2023). Recent Progress in Hyaluronic-Acid-Based Hydrogels for Bone Tissue Engineering. Gels.

[69] Szymańska E., Wojasiński M., Dąbrowska J., Krzyżowska M., Nowicka M., Ciach T., Winnicka K. (2022). Chitosan-Poly(Ethylene Oxide) Nanofibrous Mat as a Vaginal Platform for Tenofovir Disoproxyl FumarateThe Effect of Vaginal pH on Drug Carrier Performance. Int. J. Biol. Macromol..

[70] Deshkar S.S., Shirolkar S.V., Patil A.T., Mittal K.L., Bakshi I.S., Narang J.K. (2020). Vaginal Bioadhesive Drug Delivery Systems and Their Applications. Bioadhesives in Drug Delivery.

[71] Valamla B., Thakor P., Phuse R., Dalvi M., Kharat P., Kumar A., Panwar D., Singh S.B., Giorgia P., Mehra N.K. (2022). Engineering Drug Delivery Systems to Overcome the Vaginal Mucosal Barrier: Current Understanding and Research Agenda of Mucoadhesive Formulations of Vaginal Delivery. J. Drug Deliv. Sci. Technol..

[72] Brockhausen I. (2010). Biosynthesis of Complex Mucin-Type O-Glycans. Comprehensive Natural Products II.

[73] Tuğcu-DemiRöz F. (2017). Development of in Situ Poloxamer-Chitosan Hydrogels for Vaginal Drug Delivery of Benzydamine Hydrochloride: Textural, Mucoadhesive and in Vitro Release Properties. Marmara Pharm. J..

[74] Aka-Any-Grah A., Bouchemal K., Koffi A., Agnely F., Zhang M., Djabourov M., Ponchel G. (2010). Formulation of Mucoadhesive Vaginal Hydrogels Insensitive to Dilution with Vaginal Fluids. Eur. J. Pharm. Biopharm..

[75] Babelyte M., Peciulyte L., Navikaite-Snipaitiene V., Bendoraitiene J., Samaryk V., Rutkaite R. (2023). Synthesis and Characterization of Thermoresponsive Chitosan-Graft-Poly(N-Isopropylacrylamide) Copolymers. Polymers.

[76] Peppas N.A., Huang Y. (2004). Nanoscale Technology of Mucoadhesive Interactions. Adv. Drug Deliv. Rev..

[77] Cook M.T., Khutoryanskiy V.V. (2015). Mucoadhesion and Mucosa-Mimetic Materials—A Mini-Review. Int. J. Pharm..

[78] Haugstad K., Håti A., Nordgård C., Adl P., Maurstad G., Sletmoen M., Draget K., Dias R., Stokke B. (2015). Direct Determination of Chitosan–Mucin Interactions Using a Single-Molecule Strategy: Comparison to Alginate–Mucin Interactions. Polymers.

[79] Campaña-Seoane M., Peleteiro A., Laguna R., Otero-Espinar F.J. (2014). Bioadhesive Emulsions for Control Release of Progesterone Resistant to Vaginal Fluids Clearance. Int. J. Pharm..

